# Structure and Nonlinear Spectra of the Basal Face of Hexagonal Ice: A Molecular Dynamics Study

**DOI:** 10.3390/molecules30173619

**Published:** 2025-09-04

**Authors:** Konstantin S. Smirnov

**Affiliations:** Univ. Lille, CNRS, UMR 8516— LASIRe—Laboratoire Avancé de Spectroscopie pour les Interactions la Réactivité et l’Environnement, F-59000 Lille, France; konstantin.smirnov@univ-lille.fr

**Keywords:** hexagonal ice, basal face, quasi-liquid layer, structure, second-order susceptibility, nonlinear spectra, molecular dynamics simulations

## Abstract

Structure and nonlinear spectra of the basal surface of ice Ih were investigated by molecular dynamics simulations. At a temperature significantly lower than the melting temperature $T_{m}$, the ice structure at the interface is only weakly perturbed by the presence of surface. The computed nonlinear spectrum of the interface well agrees with the experimental data and the results of the calculations provide the molecular-level interpretation of spectral features. In particular, the ice surface specific positive peaks in the Im[$\chi^{\left(2\right)}$] spectrum at ∼3180 cm^−1^ and at ∼3420 cm^−1^ were found to result from the low- and high-frequency vibrational modes of quadruply H-bonded surface molecules, respectively. The spectrum of the crystalline ice interface is significantly affected by intermolecular interactions. Upon increasing the temperature, the structural disorder extends to the second water bilayer. The thickness of the premelted water layer of 6–8 Å can be estimated at the temperature by ca. 5 K below $T_{m}$. The increase in the temperature results in a change in the intensity and shape of the nonlinear spectrum of the ice Ih interface. The changes can be explained by the interconversion between different H-bonded surface species and by an increase in disordering of water molecules that reduces strength of intermolecular interactions. Results of the present work contribute to our understanding of the structure–spectrum relationship of the ice/air interface, and shed light on the origins of features in the nonlinear spectra of the system.

## 1. Introduction

Ice is likely one of the most important solids. In interstellar space, it can be found as a component of comets, in some planets, and in icy moons. On Earth, ice plays an important role in many atmospheric, geophysical, and (geo)chemical processes [1,2,3]. Ice has a very rich phase diagram and, currently, more than twenty ice phases are known, including amorphous modifications, with the hexagonal ice Ih being the most abundant phase under ambient conditions [4,5]. Physico-chemical processes occurring on the surface of ice play a crucial role for a multitude of phenomena at different scales, ranging from the glacier motion to the chemical reactions of small molecules with ice particles in the atmosphere [3,6]. Consequently, the structure of the ice surface and its change with temperature remain a subject of intensive research.

Experimental investigations of ice surfaces encounter two major problems. The first one, common to many surface studies, is disentangling the response of the surface from that of the bulk. The second problem, related to a fragile nature of ice surface, is that the employed experimental technique should be non-invasive, thereby avoiding the modification of the surface upon the interaction with the probe. The first problem is commonly overcome by surface-specific methods, such as scanning probe techniques (AFM, SFM, STM), X-ray reflectivity, and spectroscopic methods (XPS and nonlinear sum-frequency generation (SFG) spectroscopies) [7]. Among the latter techniques, vibrational sum-frequency generation (VSFG) spectroscopy is of particular use because the method obviates the second difficulty and it provides a molecular-level information about species present on the surface [8,9]. A review of advances in VSFG studies of ice surfaces and interfaces can be found in refs. [10,11].

The quantity responsible for the nonlinear response measured with the SFG spectroscopies is the second-order nonlinear susceptibility $\chi^{\left(2\right)}$ that differs from zero because the presence of surface breaks the inversion symmetry characteristic of most bulk structures [8,12]. VSFG studies are carried out with either conventional (homodyne-detected) setups, which give a spectrum of the $|\chi^{\left(2\right)}{|}^{2}$ quantity, or with heterodyne-detected (HD-VSFG) setups yielding both the imaginary and real components of the complex $\chi^{\left(2\right)}$ quantity. In the latter case, the analysis of the sign of features in the Im[$\chi^{\left(2\right)}$] spectrum can provide a structural information about the surface species. However, extracting this information solely from the experiments is a highly complex task that makes atomistic simulations indispensable for the understanding and interpretation of the spectroscopic data [13,14].

Since 1850, ice surfaces are known to have a thin layer of premelted water, often referred to as the quasi-liquid water layer (QLL) [15]. However, the thickness of the QLL, its structure, and its change with temperature are still under debate. The current understanding of the premelted water layer as well as the outlook of QLL research have been presented in refs. [16,17,18]. Pioneering VSFG studies of the basal (0001) ice Ih surface by Shen and co-workers showed an onset temperature of about 200 K for surface melting of the Ih structure [19,20,21]. This temperature, which is of ca. 70 K below the ice melting temperature $T_{m}$, is notably lower than the onset of surface melting obtained with the X-ray scattering technique [22]. In their work, Shen and co-workers paid particular attention to the spectral peak at about 3700 cm^−1^ due to the free OH bonds of surface H_2_O molecules [19]. The continuous behavior of an orientation order parameter of the OH bonds with temperature, contrasts with the expected step-wise change typical of the first-order phase transition, such as bulk melting. Above the onset temperature, the structure of the surface layer was found to become increasingly disordered. Shen and co-workers concluded that the QLL has a temperature-dependent structure with structural variations across the layer.

An important breakthrough in the understanding of ice surface melting was presented by Sánchez et al. [23], who observed a step-wise blue shift of the position of hydrogen-bonded OH stretch peak in the VSFG spectra of the basal ice surface at 257 K. Below this threshold temperature, the spectra did not show a notable dependence on temperature, although the premelted water layer was found to be already present on the surface. From the noncontinuous behavior of the band position, the authors concluded that QLL suddenly increases its thickness around 257 K and a comparison of the experimental findings with results of MD simulations allowed the bilayer-by-bilayer mechanism of surface ice melting to be suggested. Sánchez et al. [23] pointed out that QLL seems to be more similar to ice than to supercooled liquid water, with stronger H-bonds than liquid water. Nagata et al. [24] reviewed the progress of the experimental and simulation studies probing the ice/air interface and presented a unified view on different states of QLL. Their combined experimental/computational study revealed that the topmost layer of ice surface is disordered already at 183 K, while the second layer becomes disordered at 257 K. The disorder causes the conversion of triply hydrogen-bonded surface water molecules to a doubly H-bonded species that accounts for the blue shift of the free-OH band in the VSFG spectra as the temperature rises above 183 K.

Shultz and co-workers investigated the basal plane of hexagonal ice in a temperature range of 113–178 K, below the presumed onset temperature of surface melting. The comparison of spectra measured with different polarizations showed that the ppp and ssp VSFG spectra are two orders of magnitude more intense than the sps one, and that the spectral intensity increases with decreasing temperature [25]. The spectra were dominated by a peak at 3100 cm^−1^ and the ppp spectrum contained more details. The high spectral intensity of the ice surface compared with that of the liquid water/air interface and the observed intensity variation with the temperature were attributed by the authors to cooperative dynamics whose extent increases with decreasing temperature. This explanation was corroborated by results of Morita and co-workers [26]. Making use of quantum mechanics/molecular mechanics simulations, they explained the high spectral intensity of the ice/air interface by “bilayer-stitching” modes characterized by a significant intermolecular charge transfer. A polarization analysis SFG study by the Shultz group showed that the dependence of the ppp spectrum on polarization can be reproduced with at least five different oscillators [27]. A following computational study by the same authors suggested that the ppp VSFG spectra of the basal face of ice Ih at 128 K better corresponds to a phase that alternates rows of H and O atoms lacking an H-bond, rather than to a proton disordered surface [28].

Bakker and co-workers studied the signatures of the OH stretch vibrations of the basal face of ice Ih as a function of temperature by the HD-VSFG technique and molecular dynamics simulations [29,30,31]. The spectral response of the surface at 245 K was found to contain a component that was very similar to the spectrum of super-cooled liquid water [29]. At a temperature of 150 K, the authors distinguished seven contributions in the Im[$\chi^{\left(2\right)}$] spectrum, five of which have a counterpart in the bulk response, and two of which are the surface-specific modes with frequencies of 3530 cm^−1^ and 3700 cm^−1^ [30]. The feature at 3530 cm^−1^ was not reported previously and, using the MD simulations, it was found to contain contributions from OH stretch vibrations of interfacial molecules forming four H-bonds and of molecules with two donor and one acceptor hydrogen bonds. The peak at 3700 cm^−1^ due to free OH bonds of surface H_2_O molecules revealed a temperature dependence, which was explained by the interconversion of different H-bond donating and H-bond accepting molecules on the surface [31]. Bakker and co-workers concluded that the topmost monolayer of water molecules has a minimum in free O-H groups and a maximum in hydrogen bonds around 200 K [31].

Yamagushi and co-workers measured the Im[$\chi^{\left(2\right)}$] spectra of three main faces of ice Ih at T=130 K and found that the signal is essentially generated at the surfaces [32]. Since the bulk structure of ice Ih is proton-disordered, the authors concluded that the SFG response is due to a surface proton ordering along the normal to the surface. Consequently, an observed strong positive peak at 3100 cm^−1^ was attributed to an “H-up” surface proton order, where the hydrogen atom of OH groups point outwards the bulk. Subsequent study of the isotopically diluted ice Ih by the same group consolidated the conclusion [33], while no explanation of the origin of the peculiarity was put forward.

As was mentioned before, atomistic simulations have often been used to account for experimental results [14,24,26,28,30,31,33]. Very recently, two computational studies reported on the VSFG spectra of ice/air interface [34,35]. Berrens et al. [34] computed the nonlinear spectra of the proton-ordered (ice XI) and disordered (ice Ih) ice surfaces at a temperature of 100 K lower than melting temperature. The MD simulations, which used an ab initio trained machine-learning (ML) potential, demonstrated differences between the surfaces of the two structures in both the $|\chi^{\left(2\right)}{|}^{2}$ and the Im[$\chi^{\left(2\right)}$] spectra. Analysis of the spectra indicated that the nonlinear response of crystalline ice surfaces mainly resulted from the top two bilayers, regardless the ice structure. Making use of the vibrational density of states of H atoms of different surface species, the authors provided a molecular-level assignment of features in the computed spectra.

The modeling study by Rashmi et al. [35] was carried out with the MB-pol potential [36,37,38,39] and an MB-pol-based ML potential [40] with and without considering nuclear quantum effects (NQE). The VSFG spectra of the ice/air interface calculated at different temperatures generally agreed with experimental results, except for a low-frequency peak appearing in the experimental spectra at low temperatures. Analysis of the computed spectra revealed spectral features due to individual monolayers of water molecules, related to hydrogen-bonding configurations that govern the formation of QLL. The simulations showed that the formation of QLL begins at a temperature of ca. 60 K below the melting point and the melting proceeds in the bilayer-by-bilayer manner, in line with results by Sánchez et al. [23]. This process was characterized by distinct frequency shifts of the OH stretching modes of water molecules within the topmost bilayer.

It should be noted that few experimental HD-VSFG spectra of the ice Ih/air interface show some discrepancies. Thus, the spectra measured by the group of Bakker featured a low-intense positive band at ca. 3080 cm^−1^ and a strong negative band at ca. 3150 cm^−1^, in contrast to the strong positive peak at 3090 cm^−1^ and a low-intensity broad negative feature at about 3200 cm^−1^ observed in the Im[$\chi^{\left(2\right)}$] spectra by Yamagushi and co-workers [32,33]. The latter authors discussed possible reasons of the disparity in detail and ascribed this π/2 phase shift in the spectra to differences in the experimental setups used by the two research groups [10]. From the computational side, making use of the effective vibrational Hamiltonian, Yamagushi and co-workers have successfully reproduced their experimental Im[$\chi^{\left(2\right)}$] spectra [33]. On the other hand, the outcome of MD simulations reported in refs. [14,26,30,34,35] seems to be in a better agreement with the spectra measured by the Bakker group, although the recent modeling studies fail to mimic the characteristic low-frequency positive feature in the spectrum. Comparison of simulation results between each other is often hampered by differences in models and computational procedures.

The present work reports results of a molecular dynamics study of the structure and nonlinear spectra of ice Ih surfaces. It focuses on the basal face of the hexagonal ice as the majority of the experimental and computational results concerns this system. The study addresses a modification of surface structure with temperature and it proposes the molecular-level interpretation of features in the HD-VSFG spectra. The work also attempts to explain the mentioned inconsistencies in the experimental results and it completes the previous modeling studies of the system, thereby extending our knowledge of the ice surfaces.

## 2. Results and Discussion

### 2.1. Ice/Air Interface at ΔT=−65 K

**Figure 1:**
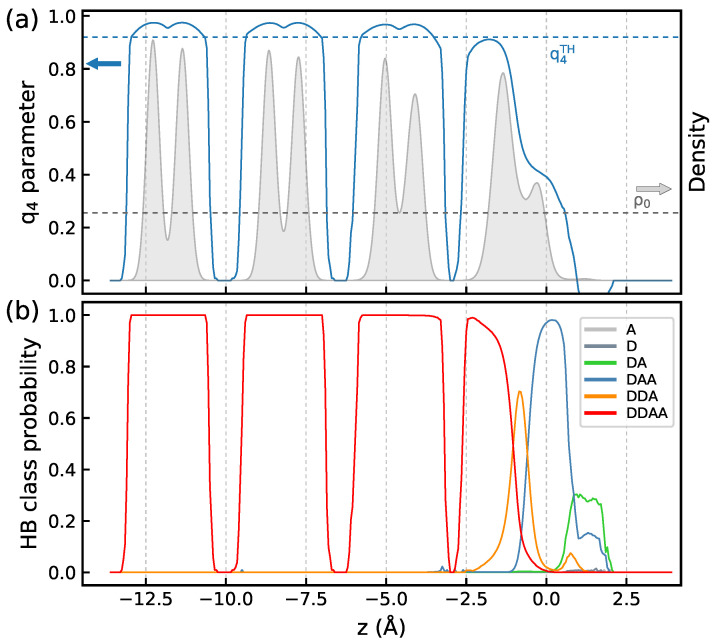
(**a**)—*z*-profiles of water density $\rho^{*}$ and of the $q_{4}$ order parameter (4), (**b**)—*z*-profiles of H bonded species in the first four bilayers of the Ih/air interface at ΔT=−65 K. The dashed horizontal lines in the panel (**a**) indicate the $q_{4}^{TH}$ and $\rho^{*}=1$ values.

We begin our analysis with the Ih/air interface at ΔT=−65 K. Figure 1a presents the *z*-profiles of the density $\rho^{*}$ and of the $q_{4}$ order parameter. At this temperature, the ice structure is preserved across the entire interface and the structural characteristics differ from their bulk values only in the topmost water monolayer. The profiles of the H-bonded species in the system are displayed in Figure 1b and they show the presence of water molecules lacking either one donor or one acceptor H-bond in the topmost bilayer with the latter species being more distant from the surface. Molecules recover the four-fold H-bond coordination typical of bulk ice already in the second monolayer of the first bilayer. One can also notice the presence of a small quantity of doubly H-bonded DA molecules at the very top of the surface.

**Figure 2:**
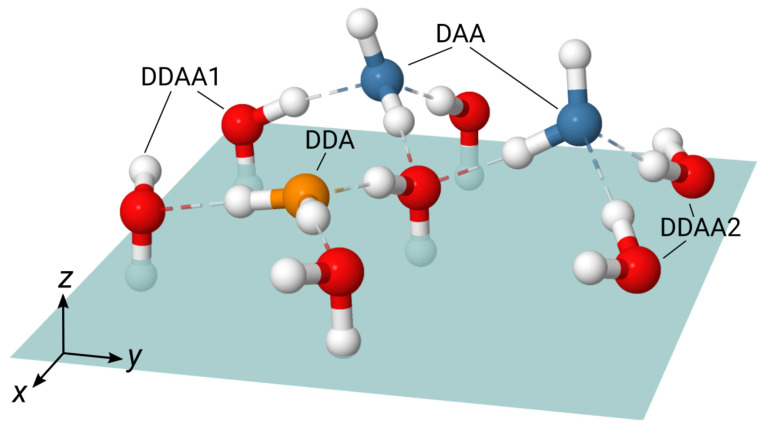
H-bonding situations of water molecules in on the surface of the Ih/air interface. The oxygen atoms are colored according to the scheme used in Figure 1b, see text for detail. Dashed lines stand for hydrogen bonds. Shaded rectangle represents the surface plane.

Figure 2 illustrates types of hydrogen bond environment of H_2_O molecules on the ice surface. It is worth noting two types of the quadruply hydrogen bonded DDAA molecules that differ by the orientation of OH bonds with respect to the normal to the surface. The first type, DDAA1, has one OH bond directed along the *z*-axis towards bulk, while the second bond is close the the surface plane and it is tilted to the positive direction of the *z*-axis. Molecules of the second DDAA type, DDAA2, have both OH bonds oriented in the positive direction of the *z*-axis, with the HH vector lying close to the surface plane (Figure 2).

**Figure 3:**
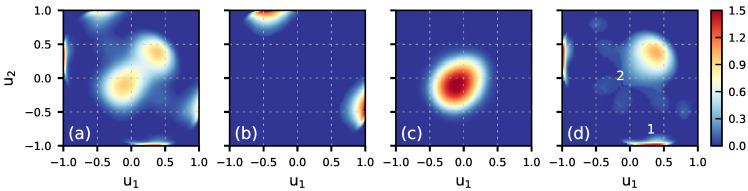
$D_{Z}\left(u_{1},u_{2}\right)$ maps for H_2_O molecules in the first bilayer (1BL) of the Ih/air interface at ΔT=−65 K. (**a**)—all H_2_O molecules in 1BL, (**b**)—DAA species, (**c**)—DDA species, and (**d**)—DDAA species. Labels ‘1’ and ‘2’ in the panel (**d**) indicate patterns due to molecules of the DDAA1 and DDAA2 subclasses, respectively.

Figure 3 displays the $D_{Z}\left(u_{1},u_{2}\right)$ (3) maps for molecules in the first bilayer and the maps for the DAA, DDA, and DDAA species. The comparison of the map for the first bilayer with that for the bulk region of the system (Figure S3a) indicates a weak orientational disorder of molecules on the surface. Regarding the orientation of molecules in the different H-bonded classes, the DAA molecules are characterized by OH bonds with the $\left[u_{1}\approx 1.0,u_{2}\approx -0.4\right]$ values that reflects a prevalent orientation with one OH bond along the positive direction of the *z*-axis and the second one towards the bulk region. The DDA molecules have both OH bonds close to the surface plane with $\left[u_{1}\approx u_{2}\approx -0.1\right]$. The $D_{Z}\left(u_{1},u_{2}\right)$ map for the DDAA class results from the two types of such molecules with the orientations given by $\left[u_{1}\approx 0.4,u_{2}\approx -1.0\right]$ and $\left[u_{1}\approx u_{2}\approx 0.4\right]$ for the DDAA1 and DDAA2 subclasses, respectively. In general, the $D_{Z}\left(u_{1},u_{2}\right)$ maps in Figure 3 are in line with the configurations seen in Figure 2.

Making use of Figure 3 and assuming the validity of the local-mode approximation, one can attempt to prefigure the sign and, to lesser extent, position of the contribution of the H-bonded classes to the Im[$\chi_{ssp}^{\left(2\right)}$] spectrum of the water layer. For instance, the DAA molecules can be expected to yield a high-frequency positive signal due to the free OH bond and a low-frequency negative band by the second bond with the H-down atom engaged in hydrogen bond with an underneath molecule, as shown in Figure 2. In a similar way, the DDA molecules are supposed to produce a weak negative signal in the spectral range typical of the O–H stretching modes of H-bonded water molecules. It should be mentioned that such an analysis refers to the part of the Im[$\chi_{ssp}^{\left(2\right)}$] spectrum which results from the self-part of the $\chi^{\left(2\right)}$ susceptibility.

**Figure 4:**
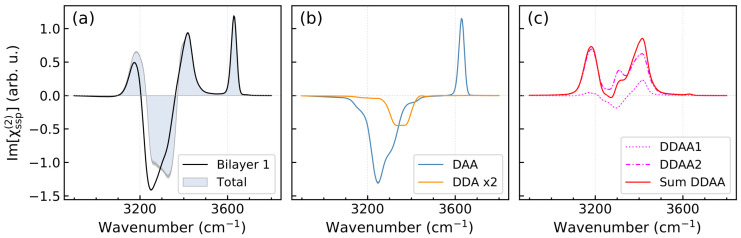
Self-part of the Im[$\chi_{ssp}^{\left(2\right)}$] spectra of the Ih/air interface at ΔT=−65 K. (**a**)—Black solid line shows the spectrum of the first bilayer, shaded contour is the spectrum of the interface computed for the maximum probing depth. Panels (**b**,**c**) display contributions of the H-bonded species (Figure 2) to the spectrum of the first bilayer.

Figure 4 presents the Im[$\chi_{ssp}^{\left(2\right)}$] spectrum of the self-part of the nonlinear susceptibility computed for the first bilayer and the contributions of molecules of the different H-bonded classes to the spectrum. One sees that the spectra of the DAA and DDA species well agree with the predictions based on the analysis of the $D_{Z}\left(u_{1},u_{2}\right)$ maps in Figure 3b,c. The spectrum of the DDAA molecules is the sum of contributions of the DDAA1 and DDAA2 subclasses. Spectrum of molecules of the former class (Figure 4c) features both positive and negative peaks due to the H-up and H-down bonds of the DDAA1 molecules, whereas the spectrum of the DDAA2 molecules has only positive signal because their both their OH bonds point in the *z*-positive direction, as shown in Figure 2 and Figure 3d. Finally, Figure 4a displays the self-part of the Im[$\chi_{ssp}^{\left(2\right)}$] spectrum computed for the maximum probing depth of 28 Å (eight water bilayers). The comparison indicates that the spectrum of the two topmost bilayers accounts for the major part of the total nonlinear response of the interface (see also Figure S4), in agreement with results of refs. [32,34,35].

**Figure 5:**
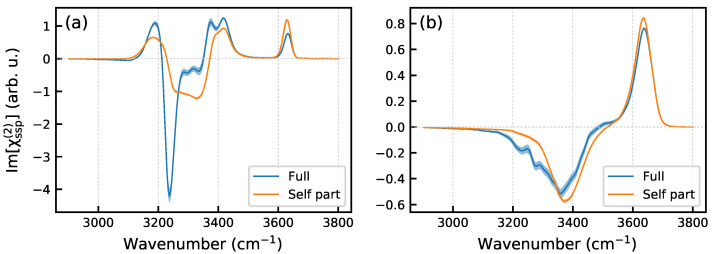
Full and self-part Im[$\chi_{ssp}^{\left(2\right)}$] spectra. (**a**)—Ih/air interface at ΔT=−65 K, (**b**)—liquid water/air interface at T=293 K. Shaded areas represent the statistical uncertainty.

As was mentioned above, the spectra shown in Figure 4 do not take into account correlations between dipoles and polarizabilities of different molecules. Figure 5a displays the Im[$\chi_{ssp}^{\left(2\right)}$] spectra of the Ih/air interface at ΔT=−65 K obtained from the full TCF and with its self part. For the sake of comparison, Figure 5b also presents such spectra computed for the water/air interface at 293 K using the same simulation protocol. Disorder in the liquid water structure and the thermal motion of H_2_O molecules result in a weak intermolecular dipole–polarizability couplings that only slightly affect the nonlinear spectrum, essentially in a low-frequency part, Figure 5b. This outcome is in line with results of previous computational studies of the system [11,41,42,43,44]. In contrast, the intermolecular correlations in the crystalline ice structure modify the spectral intensity and shape of the $\chi^{\left(2\right)}$ spectrum (Figure 5a), especially in the region below 3400 cm^−1^. In particular, the correlations significantly increase the intensity of the negative peak at ca. 3240 cm^−1^. The finding aligns with the results by Ishiyma and Morita [14,26], who attributed the high intensity of this peak to the collective dynamics of the ordered ice lattice, enhanced by charger-transfer effect. Previously, Car and co-workers [45] and Skinner and co-workers [46] have demonstrated that the intermolecular dipole interactions in ice significantly influence its linear spectra. These correlations were found to affect the entire IR spectrum, enhancing the system’s response at certain frequencies while attenuating it at others [45]. Skinner and co-workers pointed to that the strength of the interactions depends not only on the distance between two dipoles, but also on their relative orientations [46]. It is, therefore, little wonder that the intermolecular correlations manifest themselves in the nonlinear spectrum of ice surface and affect the spectrum to a greater extent, compared with the liquid water/air interface.

The decomposition of the Im[$\chi_{ssp}^{\left(2\right)}$] spectrum shown in Figure 4 allows the interpretation of spectral features in terms of H-bonding situation of molecules at the interface. The high-frequency positive peak at 3630 cm^−1^ originates exclusively from the free-OH mode of the DAA molecules. The positive feature between 3400 cm^−1^ and 3500 cm^−1^ can be assigned to contributions of the high-frequency modes of H-up bonds of the DDAA1 and DDAA2 molecules. The intense negative signal in the region 3200 cm^−1^ to 3400 cm^−1^ is due to several contributions. The major one comes from the low-frequency mode of the H-down bonds of the triply hydrogen-bonded DAA molecules and the signal is amplified by a weak contribution of the DDA molecules on the high-frequency side. Next in this region is a negative signal of the low-frequency mode of the DDAA1 species that is attenuated by a positive signal of the DDAA2 molecules so that both the contributions almost cancel each other. Finally, the positive peak below 3180 cm^−1^ is due to the low-frequency mode of the DDAA2 molecules. Thus, the positive peaks at 3180 cm^−1^ and 3420 cm^−1^ which differentiate the Im[$\chi_{ssp}^{\left(2\right)}$] spectrum of ice surface from that of the water/air interface, cf. Figure 5a,b, result from the four-fold hydrogen bonded DDAA molecules.

This interpretation is in line with the outcome of previous studies. Smit et al. [30] analyzed the origins of the positive peak at 3530 cm^−1^ in the Im[$\chi_{ssp}^{\left(2\right)}$] spectrum of the ice surface (counterpart of the 3420 cm^−1^ peak) and found that the feature is due to OH stretching vibrations of DDAA and DDA interfacial molecules. Berrens et al. [34] also ascribed the spectral intensity in the region 3450 to 3600 cm^−1^ to the O–H stretching modes of the DDAA and DDA molecules, and indicated that the overall positive intensity indicated a stronger contribution of modes localized on the OH bonds of the DDAA molecules with the H-up orientation. The signal below 3450 cm^−1^ was attributed by these authors to O–H modes of the DAA and DDAA species with a dominant contribution of modes localized on H-down bonds of the species. Rashmi et al. [35] examined contributions of different structural units in the two upmost surface bilayers to the Im[$\chi_{ssp}^{\left(2\right)}$] spectrum. The authors found that the DAA molecules were responsible for the positive free-OH peak at 3700 cm^−1^ and a wide negative peak below 3600 cm^−1^. Contribution of the DDA molecules appeared at 3550 cm^−1^ and had a negative sign. The four-fold H-bonded DDAA species yielded positive and negative signals in the regions above and below 3450 cm^−1^, respectively. The spectra of the DAA and DDA molecules computed in the present work also favorably compare to time-dependent VSFG spectra of water species on the liquid water/air interface computed by Kühne and co-workers [47]. In particular, the authors obtained that the Im[$\chi_{ssp}^{\left(2\right)}$] spectrum DDA molecules has a wide negative peak at waiting time $t_{w}=0$. A further comparison of the results of the present work with those of experimental and previous computational studies is provided in the following section of the paper.

### 2.2. Effect of Temperature

**Figure 6:**
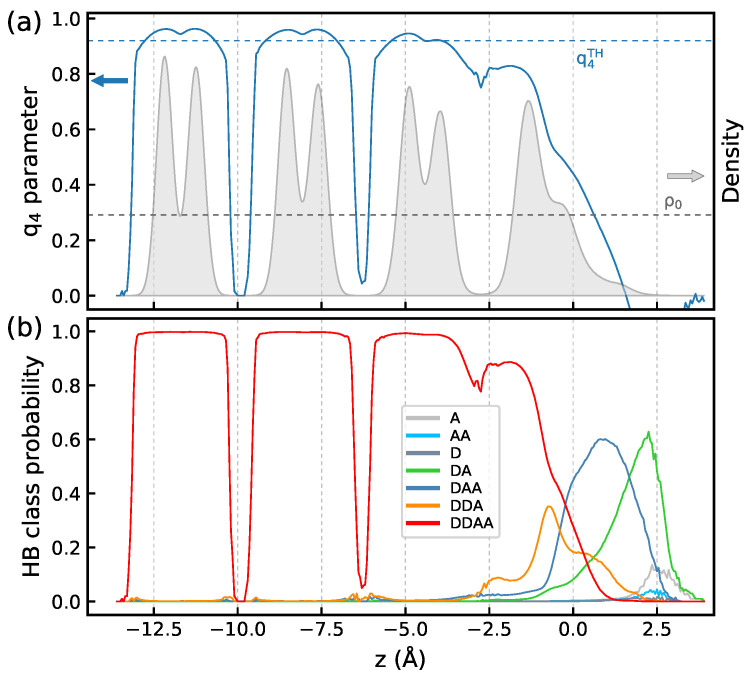
(**a**)—*z*-profiles of water density $\rho^{*}$ and of the $q_{4}$ order parameter (4), (**b**)—*z*-profiles of H bonded species in the first four bilayers of the Ih/air interface at ΔT=−5 K. The dashed horizontal lines in the panel (**a**) indicate the $q_{4}^{TH}$ and $\rho_{0}$ values.

**Figure 7:**
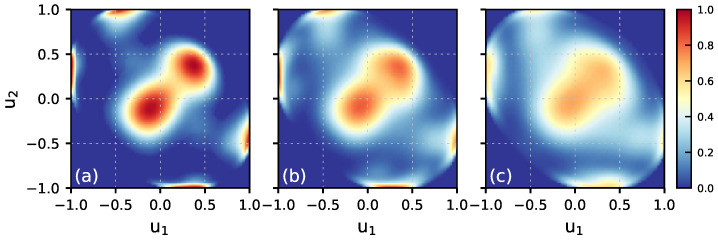
The $D_{Z}\left(u_{1},u_{2}\right)$ maps of the first bilayer of the Ih/air interface as a function of temperature: (**a**) ΔT=−65 K, (**b**) ΔT=−35 K, (**c**) ΔT=−5 K.

Figure 6a displays the *z*-profiles of the structural parameters and of the H-bonded species in the system at ΔT=−5 K, and the characteristics for ΔT=−35 K are shown in Figure S2. Upon increasing the temperature, the width of peaks for the first bilayer in the water density profile increases and the shape of the profile indicates that the topmost bilayer loses its layered structure. The tetrahedral order parameter $q_{4}$ remains below the threshold value in the entire bilayer and the increase in temperature enhances disordering of the molecular orientations in the region, as illustrated by the $D_{Z}\left(u_{1},u_{2}\right)$ maps in Figure 7. The topmost surface bilayer slightly expands upward, toward the vacuum. Compared to lower temperatures, the probability of finding doubly hydrogen-bonded DA molecules increases and small amounts of other low-coordinated H-bonded species appear on the ice surface, cf. Figure 1b and Figure 6b. However, even at the temperature close to $T_{m}$, the changes in the structure of the ice/air interface are mainly limited to the first bilayer. Indeed, the density profile and the $q_{4}$ parameter values in the second bilayer are similar to those in the bulk region of ice, Figure 6a, and the orientation of molecules in the second bilayer has only a weak dependence on temperature, Figure S3.

**Figure 8:**
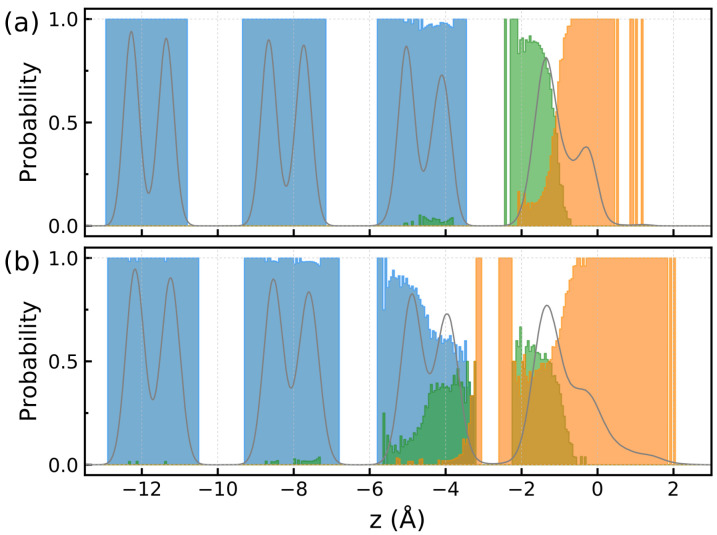
(**a**)—*z*-profiles of probability of finding a water molecule in different structures according to the LICH test [48]: (**a**) ΔT=−65 K, (**b**) ΔT=−5 K. Color legend: blue—crystalline phase, green—interfacial crystalline phase, orange—amorphous (liquid) phase, gray solid lines in the panels show the water density profile.

Figure 8 displays *z*-profile of the probability of finding water molecules in different structural units according to the LICH-test algorithm [48]. At ΔT=−65 K, the first bilayer consists of the interfacial ice and liquid water phases, whereas water molecules of the second bilayer can be found predominantly in the bulk ice environment and in a small amount of interfacial ice units (Figure 8a). When the temperature rises, the heterogeneity of the second bilayer increases via the formation of interfacial ice structures and the liquid water units appear in the upper part of the bilayer. Close inspection of Figure 8 shows that the interfacial ice units can now be detected in the third bilayer. Therefore, the structurally heterogeneous region extends over one and two water bilayers at ΔT=−65 K and ΔT=−5 K, respectively. The thickness of the premelted layer of about 6–8 Å at ΔT=−5 K agrees well with results of previous computational studies [49,50,51]. In line with results by Bishop et al. [50], the present simulations show that the disorder is limited to two surface bilayers with the topmost one having a liquid-like structure and the second bilayer remaining partially ordered.

Table 1 reports the fraction of different H-bonded water species in the first bilayer of the ice Ih/air interface. At the low temperature, the surface is populated by DAA and DDA species in equal proportions. Upon increasing the temperature, the fractions of both classes of triply hydrogen-bonded H_2_O molecules decrease to a comparable extent, while the number of doubly H-bonded water molecules increases. These are dominated by molecules of the DA class, although small amounts of AA and DD species emerge on the surface. Given the *z*-profiles of the doubly and triply hydrogen-bonded molecules in Figure 6, it is reasonable to assume that the formation of the DA species likely proceeds via breaking of one acceptor H-bond of the DAA molecules. Such a mechanism has been put forward by Smit et al. [31] to account for a frequency shift of the free OH peak with temperature in the VSFG spectra of the ice Ih/air interface. Another interesting feature also mentioned by the authors, is that the decrease in the overall amount of the DAA and DDA molecules upon increasing the temperature, is not compensated by the increase in the doubly H-bonded species. This indicates that the triply hydrogen-bonded water molecules are partially converted to the DDAA molecules when the temperature rises. This conclusion is corroborated by the trend in the fraction of the DDAA species with temperature (Table 1).

**Figure 9:**
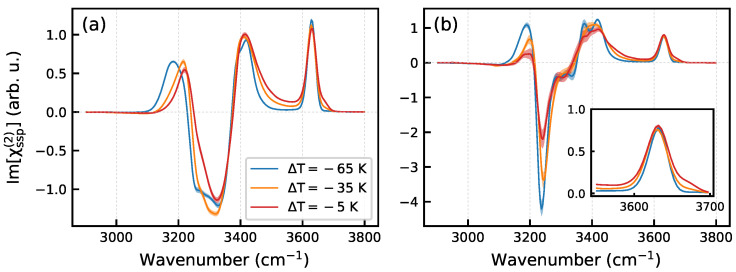
Im[$\chi_{ssp}^{\left(2\right)}$] spectra of the Ih/air interface as a function of temperature: (**a**)—self-part spectra, (**b**) full spectra. The inset in panel (**b**) shows a zoom of the 3550–3700 cm^−1^ region. Shaded areas represent the statistical uncertainty.

Figure 9 displays the spectra of the imaginary part of the self-part and of full $\chi_{ssp}^{\left(2\right)}$ quantity, as a function of temperature. The spectrum of the self part exhibits a small temperature dependence. Main changes concern (i) the positive low-frequency peak at 3180 cm^−1^ that shifts upward as the temperature increases, and (ii) a shoulder that emerges on the high-frequency side of the free-OH peak (Figure 9). The full Im[$\chi_{ssp}^{\left(2\right)}$] spectrum shows more significant changes that include, in particular, reducing the intensities of the positive and negative peaks at 3180 cm^−1^ and 3240 cm^−1^, respectively (Figure 9b). In addition, the spectrum progressively loses details as the temperature grows.

**Figure 10:**
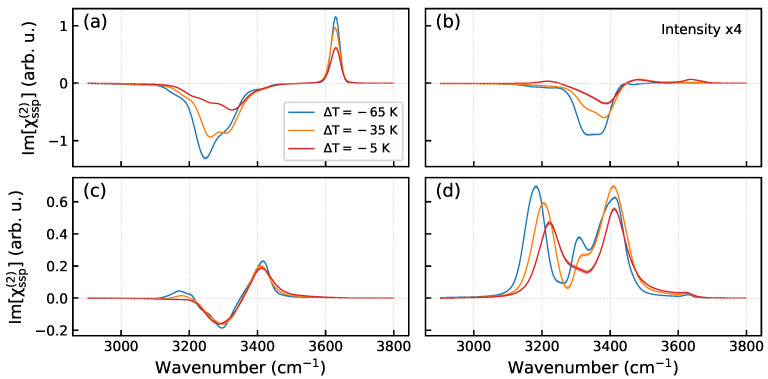
Im[$\chi_{ssp}^{\left(2\right)}$] spectra of H_2_O molecules in the H-bonded classes in the first bilayer of the Ih/air interface as a function of temperature: (**a**)—DAA molecules, (**b**)—DDA molecules, (**c**)—DDAA1 molecules, (**d**)—DDAA2 molecules. Note the difference in the *y*-axis ranges between the panels in the upper and lower row.

Figure 10 presents the decomposition of the self-part spectra shown in Figure 9a into contributions of the H-bonded species. As the temperature increases, the low-frequency peaks in the spectra of most species undergo un upward shift and decrease in intensity. The upward shift of the positive peak at ca. 3180 cm^−1^ with temperature in Figure 9 is due to the changes in the spectrum of the DDAA2 molecules (Figure 10d). It is noteworthy that the spectrum of the DDAA1 species is almost insensitive to the temperature (Figure 10c). These molecules serve as linkers between the bilayers (Figure S1b) and, because the increase in temperature has minimal impact on the orientation of molecules in the second bilayer (Figures S3 and S5), the hydrogen bonds of the DDAA1 molecules (interlayer stitching H-bonds) remain largely unaffected by the temperature increase. The high-frequency shoulder of the free-OH peak is due to the appearance of the doubly H-bonded DA molecules on the surface, Table 1 and Figure S6. The calculation show that the free-OH mode of the DA molecules has a frequency by about 34 cm^−1^ greater than the frequency of the counterpart mode of the DAA species. Previously, Skinner and co-workers found a difference of 39 cm^−1^ for the OH mode of HOD molecule in the DAA and DA classes [52] and the outcome of the present calculations agrees well with this finding. It has been proposed that the formation of the doubly hydrogen-bonded species at the surface explains the observed blue shift of the OH peak in the Im[$\chi^{\left(2\right)}$] spectra of the Ih/air interface with increasing temperature [31]. The above result corroborates this interpretation. The behavior of the spectra with the growth of temperature can be ascribed to a combined action of two effects: (i) increasing the amplitude of molecular reorientation and (ii) weakening the H-bond strength with the increase in thermal motion. These effects are discernible in Figure 7 and Figure S7, respectively. Their combined action reduces the intermolecular couplings and leads to the changes in the Im[$\chi^{\left(2\right)}$] spectra of the interface, Figure 9b.

**Figure 11:**
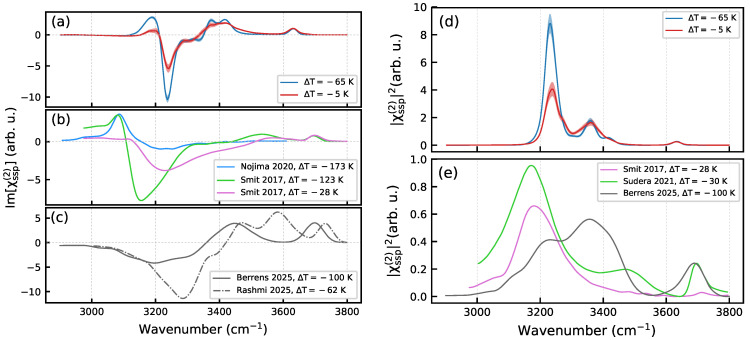
Comparison of calculated nonlinear spectra of the ice Ih/air interface with literature data. Im[$\chi_{ssp}^{\left(2\right)}$] spectra: (**a**) — present work, (**b**)—experiment [30,33], (**c**)—previous calculations [34,35]. For comparison purposes, the spectra in each panel were normalized relative to the intensity of the free-OH peak, whenever possible. $|\chi_{ssp}^{\left(2\right)}{|}^{2}$ spectra: (**d**)—present work, (**e**)—experiment and previous calculations [30,34,53].

Figure 11 compares the computed nonlinear spectra of the ice Ih/air interface with results of experimental studies and previous calculations. The shape and the temperature dependence of the modeled Im[$\chi_{ssp}^{\left(2\right)}$] spectra are consistent with the HD-VSFG spectra measured by the group of Bakker [30]. In particular, the simulations reproduce the positive peak below 3200 cm^−1^ and its behavior with temperature. The $\chi_{ssp}^{\left(2\right)}$ intensity spectra shown in Figure 11d,e are also in agreement with the measurements, especially the recent results by Backus and co-workers [53]. The main discrepancy between the calculated and the experimental spectra is that the simulations produce the spectral response in a narrower frequency interval compared with the experiments. This shortcoming can be attributed to the use of the classical SPCFw water model, which neglects the effect of the charge transfer and polarization on the O–H potential function and NQEs. While the NQEs play a minor role for the structure and stabilization of H-bonds [54], they are important for describing the dynamics of light H atoms. The magnitude of NQEs was shown to depend on hydrogen-bonding environments that cause a non-uniform red shift of OH stretching mode frequencies of molecules on the ice/air interface [35]. Interestingly, a shift of opposite sign was obtained for the OH modes of molecules in the topmost surface layer of liquid water/air interface [55]. Intermolecular charge transfer has been shown to be essential for reproducing the dipole moment of H-bonded water molecules [56,57]. It is likely that the same applies to the transition dipoles as even a small charge transfer over the distance of hydrogen bond length can yield a substantial dipole variation. Note that the scale factor presented in Appendix B takes this effect in a mean-field way. With this in mind, the ML water models employed the recent simulation studies [34,35] are expected to be superior. Indeed, the spectra computed in those works better reproduce the experimental frequency range of the O–H stretching vibrations. However, the inspection of Figure 11 shows that none of the computed spectra quantitatively reproduce both the positions and intensities of the peaks in the experimental spectra, and that the simulation results reveal a noticeable discrepancy, as do the experimental ones. Lastly, the ice surface models used in the calculations are defectless, whereas the real surfaces can have a certain amount of point and extended defects. As the nonlinear response comes from few surface layers, the presence of defects can affect the intensity of peaks and result in blurring the spectrum.

The origin of the disparity between the experimental spectra could be due to differences in the experimental setups and in sample preparation routines. The former point has already been mentioned by Yamaguchi and co-workers [10]. As to the latter, the ice sample preparation routine determines the presence and concentration of defects on the surface and the recent simulations have indicated that defects on the ice surface can play an important role for the characteristics of the premelted water layer [35].

On the modeling side, although different water models yield a similar thickness of QLL [49], the structure of the layer may slightly vary with the model thus, resulting in different nonlinear response. In addition, dipole and polarizability models used in the spectra calculation can affect the peaks intensity that further complicates the analysis of results and their comparison with experiments. Thus, the spectra in Figure 11a,c are normalized relative to the intensity of the free-OH peak. This is justified by the lack of interconversion between H-bonded species at low temperature, regardless the water model and, therefore, by the same concentration of molecules with the free OH bond on the surface. Nonetheless, the spectra in the H-bonded region reveal a marked disparity in both the shape and intensity of peaks. This discrepancy can be due to (i) a different structure of the interface with different fractions of H-bonded species and/or (ii) the use of different intensity models. The inconsistency in the results suggests that further work is needed to develop a computational approaches that can reliably produce consistent surface-specific nonlinear spectra.

## 3. Models and Computations

### 3.1. Simulation Setup

The structure of hydrogen disordered hexagonal ice was obtained with GenIce [58,59] and it consisted of 2560 H_2_O molecules arranged in 16 bilayers along the *z*-axis perpendicular to the basal face (Figure S1). The structure was equilibrated in an NPT MD run of 2 ns length for a supercooling degree ΔT=−65 K (T=130 K). The last 1 ns part of the run was used to obtain equilibrium side lengths of the orthorhombic simulation box that were equal to 35.26 Å, 38.20 Å and 57.90 Å along the *x*, *y* and *z* axes, respectively. A free basal surface was created by increasing the size of the simulation box to 200 Å along the *z*-axis and the periodic boundary conditions were applied to the system in three dimensions.

### 3.2. Interatomic Potentials

Water molecules were described with the SPCFw model [60], which represents intermolecular interactions by a sum of the Lennard–Jones (12–6) and Coulomb potentials with the parameters of the SPC model [61]. The intramolecular part of the water force field was presented in detail in ref. [62]. Previously, the SPCFw model has been successfully used for studying the structure and nonlinear spectra of mineral/water interfaces [62,63,64,65]. A simulation of water/ice interface with the model has shown that the exchange of molecules between the two phases begins at about 195 K that gives an estimate of ice melting temperature $T_{m}$ for the model. This value compares well to $T_{m}=191\pm 5$ K for the original SPC model [66,67]. As the melting temperature is specific to water model, the discussion refers to a supercooling degree $\Delta T=T-T_{m}$, instead of absolute temperature value.

### 3.3. MD Simulations

Three ΔT values, equal to −65 K, −35 K, and −5 K, were used in the work. For each target value of supercooling degree, the initial model of ice/air interface was equilibrated in a 10 ns MD run in an NVT ensemble. Then, the final structure of the run was taken as an initial structure in five series of ten production runs. Each production run consisted of 50 ps equilibration period in the NVT ensemble that was followed by a 50 ps NVE stage during which the coordinates and velocities of atoms were stored each 4 fs for the last 40 ps. The last configuration of each run was used as the starting configuration of next run with atomic velocities were newly chosen from the Maxwell–Boltzmann distribution.

The classical equations of motion were integrated using the velocity form of the Verlet algorithm with the integration time-step of 0.5 fs. The real space cut-off radius for the short-range and electrostatic interactions was equal to 10 Å. The discontinuity of the short-range energy and force at the cut-off distance was corrected with a shifted-force technique. The long-range electrostatic interactions were handled using a damped shifted-force modification of Wolf method [68,69] with a damping parameter of 0.2 Å^−1^ [69]. Temperature in the simulations which employed canonical (NVT) ensemble was controlled via a chain of Nosé-Hoover thermostats [70]. The anisotropic form of the Berendsen barostat [71] was used in the NPT equilibration run.

### 3.4. Structural Characteristics

The distribution of water molecules along the direction perpendicular to the surface (*z*-axis) was characterized by a profile of relative water density $\rho {\left(z\right)}^{*}=\rho \left(z\right)/\rho_{0}$, where $\rho_{0}$ is the density of bulk liquid water at T=293 K. To portray the orientation of H_2_O molecules with respect to the *z*-axis, a two-dimensional conditional probability density $P_{Z}\left(u_{1},u_{2}\right)$ was calculated [72]:

(1) $$P_{Z}\left(u_{1},u_{2}\right)=\frac{1}{N_{Z}}\langle \sum_{i\in Z}\sum_{k\in i}\delta \left(u_{1}-\cos\phi_{1}^{\left(i\right)}\right)\cdot \delta \left(u_{2}-\cos\phi_{2}^{\left(i\right)}\right)\rangle$$

with $\cos\phi_{k}^{\left(i\right)}$ being the cosine of angle between the *k*-th OH bond vector of molecule *i* and the *z*-axis. The distribution (1) is computed for molecules in region Z and the normalization factor $N_{Z}$ in (1) is given by

(2) $$\int_{-1}^{1}P_{Z}\left(u_{1},u_{2}\right)\;du_{1}du_{2}=1.$$

The work discusses hereafter the following quantity

(3) $$D_{Z}\left(u_{1},u_{2}\right)=\ln\left(1+P_{Z}\left(u_{1},u_{2}\right)/P_{\infty }\left(u_{1},u_{2}\right)\right),$$

where $P_{\infty }\left(u_{1},u_{2}\right)$ is the probability density distribution (1) in the isotropic medium taken as bulk liquid water at 293 K. The use of logarithmic scale in (3) permits the smoothing out of large variations of the probability density in a system with orientationally ordered/disordered regions.

A local ordering around a molecule *i* was described with the $q_{4}$ tetrahedral order parameter [73]

(4) $$q_{4}=1-C\left(n\right)\sum_{j=1}^{n-1}\sum_{k=j+1}^{n}{\left(\cos\varphi_{jik}+\frac{1}{3}\right)}^{2},$$

where the indices *j* and *k* run over n≤4 nearest neighbors of the molecule *i*, and $\varphi_{jik}$ is the angle formed by the oxygen atoms of the molecules *j*, *i*, and *k*. The coefficients C(n) are equal to $\frac{9}{4}$, $\frac{3}{4}$, and $\frac{3}{8}$ for n= 2, 3, and 4, respectively [74]. The $q_{4}$ parameter was found to be highly sensitive to angular disorder [75] and its value varies from −3 to 1 with $q_{4}=1$ for water molecule in a perfect tetrahedral environment. The parameter values calculated for the bulk Ih and supercooled water at 190 K are equal to $q_{4}^{Ih}=0.958\pm 0.001$ and $q_{4}^{SCW}=0.834\pm 0.001$, respectively. To distinguish the ice and amorphous phases, a threshold value $q_{4}^{TH}=0.92$ was defined as described in ref. [49].

### 3.5. H-Bond Analysis and H-Bonding Network

The intermolecular H-bonds in the systems were identified with the use of geometric criteria: two water molecules were considered to be hydrogen-bonded if the O–O distance was smaller than 3.3 Å and the O−H···O angle was greater than 140° [76]. The molecules were then classified into eight classes according to the number and types of H-bonds the molecule donates and accepts [77]. These eight classes are denoted by the acronyms A, D, AA, AD, DD, AAD, DDA, and DDAA, where A and D stand for the acceptor and donor H-bond, respectively. For instance, a molecule which donates two H-bonds to and accepts one H-bond from a neighbor molecule, belongs to the DDA class. Characteristics of molecules in a particular class were computed only for molecules that belonged to the class at the beginning and at the end of simulation run.

Finally, based on the H-bonding information, structural units which the H_2_O molecules are involved in were identified with the LICH-test algorithm [48]. In brief, the algorithm considers tetrahedra formed by oxygen atoms of H-bonded water molecules. Based on the connectivity information, representation matrices are computed and compared to template matrices corresponding to staggered or eclipsed conformations of the tetrahedra. Each oxygen (molecule) is then classified into cubic or hexagonal ice, clathrate, liquid water, and interfacial structures, by inspecting the staggered and eclipsed conformations of tetrahedra the atom is involved in. LICH-test probes the structural arrangement beyond the first hydration sphere and its results complete structural information derived from the analysis of the $q_{4}$ parameter. A detailed description of the algorithm can be found in ref. [48].

Analysis of the above characteristics as a function of distance from the surface provides a detailed insight into the structural organization of system. While discussing the results, the origin of the *z*-axis is placed at the *z* coordinate of the center of mass of surface bilayer.

### 3.6. Nonlinear Spectra Computation

Nonlinear spectra were computed with the time correlation function (TCF) approach [78]. The pqr component of the resonant part of the frequency-dependent nonlinear second-order susceptibility tensor $\chi^{\left(2\right)}\left(\omega \right)$ is given by

(5) $$\chi_{pqr}^{\left(2\right)}\left(\omega \right)=\frac{i\omega }{k_{B}T}\int_{0}^{\infty }dt\;e^{i\omega t}C_{pqr}\left(t\right),$$

where $C_{pqr}\left(t\right)$ is the time correlation function

(6) $$C_{pqr}\left(t\right)=\langle M_{r}\left(0\right)\cdot A_{pq}\left(t\right)\rangle .$$

The quantities $M_{r}$ and $A_{pq}$ in (6) are the *r* and pq elements of the system dipole M and polarizability A, respectively. The computation of the function (6) is discussed in Appendix A.

The dipole and polarizability models used in the work do not take into account field-induced changes in the dipole and polarizability derivatives, known as non-Condon effects [52,79,80]. The models consider the changes in a very limited extent, essentially via the mechanical anharmonicity of the O–H potential. To correct the shortcoming and to account for the environment effect, a semi-empirical scale factor was applied to the $\chi_{pqr}^{\left(2\right)}\left(\omega \right)$. The derivation of the factor is presented in Appendix B.

The work focuses on the nonlinear spectra computed for the ssp experimental geometry, where *s*, *s*, and *p* denote the polarizations of the emitted SFG radiation, visible and IR pulses, respectively. Specifically, the $\chi_{ssp}^{\left(2\right)}$ spectrum was obtained as the average of the $\chi_{xxz}^{\left(2\right)}$ and $\chi_{yyz}^{\left(2\right)}$ spectra due to the isotropy of the system in the azimuthal angle. Spectra calculated for the ppp polarization setup are reported in Supplementary Materials (Figures S8 and S9). The thickness of the ice layer used in the spectra calculations (probing depth), if not specified explicitly, was equal to a half-size of the ice slab in the *z*-direction, ca. 28 Å.

The structural characteristics and nonlinear spectra were computed by exploiting the system’s symmetry with respect to the xy plane at z=0. Special attention was given to vector quantities, such as the dipole moment, where the sign of the *z*-component was inverted for molecules with a negative *z*-coordinate of the center of mass. This approach assured that, when calculating $\chi_{pqr}^{\left(2\right)}$ with r=z, the cancellation of the *z*-component of the dipoles of molecules on the opposite surfaces of the ice slab was avoided.

## 4. Conclusions

Molecular dynamics simulations of the basal ice Ih surface show that at temperatures significantly lower than the melting temperature $T_{m}$, the structure of the ice surface is weakly perturbed for a depth of the first molecular monolayer. Interfacial water molecules form either three or four hydrogen bonds and their orientations only slightly deviate from those in the bulk ice. The computed nonlinear spectrum of the interface is in a good agreement with the experimental data that allows providing the molecular-level interpretation of features in the spectrum. The high-frequency positive peak at 3630 cm^−1^ in the Im[$\chi_{ssp}^{\left(2\right)}$] spectrum is exclusively due to the free OH bond of the triply H-bonded DAA molecules. The positive peaks at 3180 cm^−1^ and 3420 cm^−1^ result from quadruply H-bonded DDAA water molecules. The former stems from a low-frequency mode of molecules with the HH vector parallel to the surface, the DDAA2 species in Figure 2, whereas the latter is due to high-frequency modes of molecules of the DDAA class. The intense negative peak at about 3250 cm^−1^ is the sum of signed contributions of molecules in different H-bonded environments and the intermolecular couplings markedly affect the signal in this region. The spectral response of the two topmost bilayers accounts for the major part of the nonlinear spectrum of the system.

Upon increasing the temperature, the premelted water layer extends to the second bilayer. While the water density profile and the $q_{4}$ order parameter in the bilayer remain close to their bulk counterparts, the fraction of molecules in liquid-like structural units increases with temperature. The QLL thickness of about 6–8 Å can be estimated for a temperature by ca. 5 K below $T_{m}$. The increase in the temperature leads to changes in the intensity and shape of the Im[$\chi_{ssp}^{\left(2\right)}$] spectrum, in line with experiments. The thermally induced conversion of triply H-bonded DAA surface molecules to the doubly H-bonded DA species accounts for the appearance of shoulder on the high-frequency side of the free-OH peak. Other changes in the spectrum can be explained by a liquefaction of the surface water layer. The process, in addition to the intercoversion between different H-bonded species, is characterized by an increase in orientational disordering of molecules that reduces the strength of both the H-bonding and intermolecular couplings. The weakening of the intermolecular interactions impacts the intensities of the positive and negative peaks at 3180 cm^−1^ and 3250 cm^−1^, respectively. This implies that these spectral features could serve as indicators of structural changes in the topmost region of ice/air interface. The analysis of the results indicates a high sensitivity of the nonlinear spectra to subtle structural changes in the interfacial water layer.

Results of the present work shed light on the origins of features in the HD-VSFG spectra of the ice Ih surface and they agree with the outcome of the previous computational studies of the system. Ensemble of the findings contributes to our understanding of the structure–spectrum relationship of the ice/air interface. However, given the existing discrepancy in some results, further combined experimental and computational studies are necessary to advance the interpretation of experiments and, from a broader perspective, to strengthen our knowledge on the ice surfaces.

## Figures and Tables

**Table 1:** Fraction of different interfacial water species in the first bilayer of the ice Ih/air interface at different temperatures. Values in parentheses are the statistical uncertainties.

ΔT (K)	DA	DAA	DDA	DDAA
−65	0.004 (0.002)	0.115 (0.007)	0.114 (0.007)	0.766 (0.011)
−35	0.014 (0.003)	0.096 (0.004)	0.100 (0.004)	0.784 (0.005)
−5	0.025 (0.004)	0.085 (0.003)	0.091 (0.004)	0.791 (0.008)

**Figure A1:**
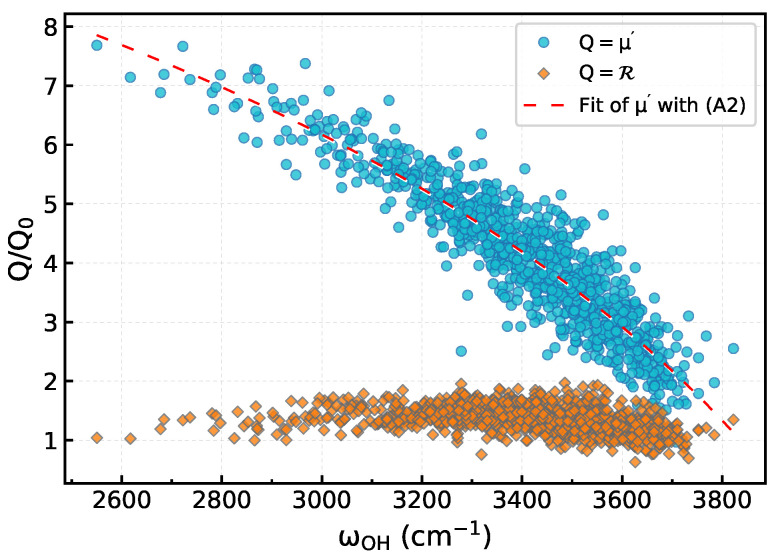
Scaled $\mu^{\prime }$ and R quantities as a function of the frequency $\omega_{\mathrm{OH}}$. The red dashed line is a fit of the $\mu^{\prime }$ data with function (A5).

## Data Availability

The raw data supporting the conclusions of this article will be made available by the author on request.

## Supplementary Materials

The following supporting information can be downloaded at: https://www.mdpi.com/article/10.3390/molecules30173619/s1, Figure S1: (a) Crystallographic cell of hexagonal ice Ih in the Cartesian frame of the simulation box shown as the shaded rectangular prism. The basal, 1^st^ prismatic and 2^nd^ prismatic faces are labeled with their Bravais-Miller indices, (0001), $\left(10\bar{1}0\right)$ and $\left(\bar{1}2\bar{1}0\right)$, respectively. (b) ice Ih structure. Atom colors: white–hydrogen, red–oxygen; oxygen atoms of one bilayer (BL) of the basal plane are highlighted with deep red color, the cyan dashed lines stand for H-bonds; Figure S2: (a) *z*-profiles of water density $\rho^{*}$ and of the $q_{4}$ order parameter, (b) *z*-profiles of H bonded species in the first four bilayers of the Ih/air interface at ΔT=−35 K. The dashed horizontal lines in the panel (a) indicate the $q_{4}^{TH}$ and $\rho^{*}=1$ values; Figure S3: $D_{Z}\left(u_{1},u_{2}\right)$ maps for H_2_O molecules. (a) bulk region of the ice Ih slab at ΔT=−5 K, (b) the second bilayer (2BL) of the slab at ΔT=−65 K, (c) 2BL at ΔT=−35 K, (d) 2BL at ΔT=−5 K; Figure S4: Im[$\chi_{ssp}^{\left(2\right)}$] spectra of the Ih/air interface at ΔT=−65 K as a function of probing depth $z_{p}$. Shaded areas represent the statistical uncertainty; Figure S5: Snapshots of the two top bilayers of the Ih/air interface: (a) ΔT=−65 K, (b) ΔT=−5 K. The oxygen atoms of the first and second bilayer are shown with deep and pale red colors, respectively, and the hydrogen atoms are in white color; Figure S6: Im[$\chi_{ssp}^{\left(2\right)}$] spectra of DDA and DA molecules on the surface of the Ih/air interface at ΔT=−5 K. Shaded areas represent the statistical uncertainty; Figure S7: Radial distribution function (RDF) of the O–O atoms pair in the first (a) and second (b) bilayers of the Ih/air interface at ΔT=−65 K and ΔT=−5 K. The zoom shows the variation of the first peak position with temperature. The shaded contour in both panels shows RDF in bulk at ΔT=−65 K; Figure S8: Im[$\chi_{pqr}^{\left(2\right)}$] spectra of the Ih/air interface at ΔT=−65 K computed for different polarizations: (a) – ssp geometry, (b) – ppp geometry. Shaded areas represent the statistical uncertainty; Figure S9: $|\chi_{ppp}^{\left(2\right)}{|}^{2}$ spectrum the Ih/air interface as a function of temperature. Shaded areas represent the statistical uncertainty. The inset shows the experimental ppp VSFG spectrum of the basal Ih surface at ΔT=−100 K [81]; Figure S10: Scaled dipole derivative $\mu^{\prime }/\mu_{0}^{\prime }$ in the O–H stretching mode(s) of H_2_O and HOD molecules, as a function of the mode frequency $\omega_{\mathrm{OH}}$. Reference [81] is cited in Supplementary Materials.

## Appendix A. Second-Order Susceptibility Calculations

The dipole and polarizability of the system are obtained as the sums of dipoles μ and polarizabilities α of individual molecules. Then, the time correlation function in (6) for the $\chi_{pqr}^{\left(2\right)}$ element reads

(A1) $$C_{pqr}\left(t\right)=\langle M_{r}\left(0\right)\cdot A_{pq}\left(t\right)\rangle =\langle \sum_{i}\mu_{r,i}\left(0\right)\cdot \sum_{j}a_{pq,j}\left(t\right)\rangle .$$

which can be recast to

(A2) $$C_{pqr}\left(t\right)=\langle \sum_{i}\mu_{r,i}\left(0\right)\cdot a_{pq,i}\left(t\right)\rangle +\langle \sum_{i}\mu_{r,i}\left(0\right)\cdot \sum_{j\neq i}a_{pq,j}\left(t\right)\rangle ,$$

where the first and second terms in (A2) are intramolecular (self) and intermolecular (cross) parts of the full TCF $C_{pqr}\left(t\right)$, respectively [82,83,84]. The calculation of the intermolecular term was performed for molecules *j* at a mean distance from the molecule *i* less than 5.5 Å which corresponds to the position of the second minimum in the oxygen-oxygen radial distribution function in bulk liquid water. The TCFs were computed on the length N=2048 and multiplied by a Hann apodization function of width N/2 prior to performing the Laplace transform in (5). The resulting $\chi^{\left(2\right)}$ spectra were smoothed by using a Gaussian filter with parameter σ equal to twice the frequency-step in the spectra (4.07 cm^−1^). The statistical uncertainty of spectral intensity was estmated by performing a bootstrap sampling of the dataset of 50 computed spectra.

To obtain the $\chi_{pqz}^{\left(2\right)}$ spectra for a specific probing depth $z_{0}$, the dipoles $\mu_{z,i}\left(0\right)$ in (A1) were multiplied by a damping function g(z)

(A3) $$g\left(z\right)=\frac{1}{2}\mathrm{sign}\left(z\right)\left(\tanh(s(|z|-z_{0}))+1\right),$$

where $|g(z_{0})|=1/2$ and the parameter *s* determines the width of transition region [83,84]. The used value of s=8.8 Å^−1^ gives the width of ∼0.25 Å for the 0.1–0.9 region and the thickness of the transition region allows separate contributions of water bilayers of the ice structure to the spectra. The use of the sign() function in (A3) avoids the cancellation of the *z*-component of molecular dipoles in the *z*-positive and *z*-negative parts of the ice slab.

## Appendix B. Frequency-Dependent Scale Factor for *χ*^(2)^

The nonlinear susceptibility $\chi^{\left(2\right)}$ of a molecular system can be approximated by the sum of first-order hyperpolarizabilities β of individual molecules [13,85]. A perturbation theory expression for the pqr element of β reads [13,78,85,86]

(A4) $$\beta_{pqr}\approx \sum_{k}\frac{\mu_{r}^{\prime }\;\alpha_{pq}^{\prime }}{\omega_{k}-\omega -i\Gamma_{k}},$$

where the sum runs over normal modes *k*, and $Q_{k}$, $\omega_{k}$, and $\Gamma_{k}$ denote the *k*-th normal mode coordinate, mode’s frequency, and damping parameter, respectively. In (A4), $\mu_{r}^{\prime }$ and $\alpha_{pq}^{\prime }$ stand for the *r* and pq components of the molecular dipole and polarizability derivatives with respect to the coordinate $Q_{k}$, respectively. These derivatives, the transition dipole and transition polarizability in quantum ansatz, account for the intensity of bands in the IR and Raman spectra.

The formation of hydrogen bonds between an H_2_O molecule and its neighboring molecules influences not only the vibrational transition energies, but also alters the magnitude of the corresponding transition dipole moments and transition polarizabilities. Quantum-chemical calculations indicate that both polarization and intermolecular charge transfer significantly contribute to the modulation of the O–H stretching transition dipole in hydrogen-bonded systems while they have a smaller influence on the transition polarizability [79,85,87]. These effects are overlooked in the dipole and polarizability models employed in the work. Consequently, a frequency-dependent scale factor is introduced to account for the environment effect.

Dependencies of the dipole and polarizability derivatives on the frequency $\omega_{\mathrm{OH}}$ of OH stretching mode for an H-bonded water molecule were obtained in the following way. A classical MD simulation of bulk liquid water (864 SPCFw H_2_O molecules in a cubic box at the experimental density) was carried out for a temperature of 293 K and a set of 500 molecular clusters was extracted from the simulation trajectory. Each cluster consisted of a central molecule surrounded by molecules in a sphere of 5.6 Å radius (two hydration spheres). These clusters were used in quantum-chemical DFT calculations using GAUSSIAN 16 program [88]. The central molecule and its four nearest neighbors were treated at the B3LYP/6-311++(d,p) level. More distant molecules were considered at the B3LYP/6-31G level. The central molecule underwent a full structural optimization followed by the vibrational analysis, whereas the atoms of other molecules were held fixed. The Hessian matrix and Cartesian derivatives of the dipole and polarizability were then used to obtain the frequencies $\omega_{\mathrm{OH}}$, and $\mu^{\prime }$ and $\alpha^{\prime }$ values of the OH stretching modes. The effect of H-bonding on $\alpha^{\prime }$ was evaluated by computing $R={\left[45{\left({\bar{\alpha }}^{\prime }\right)}^{2}+7{\left(\gamma^{\prime }\right)}^{2}\right]}^{1/2}$ as a function of $\omega_{\mathrm{OH}}$, where ${\bar{\alpha }}^{\prime }$ and $\gamma^{\prime }$ are the isotropic part and the anisotropy of the $\alpha^{\prime }$ tensor, respectively. The quantity $R^{2}$ is reported by GAUSSIAN as Raman activity. Finally, the vibrational frequencies in quantum-chemical calculations are known to be overestimated compared with the experimental values. To correct the systematic error, the computed frequencies were multiplied by a coefficient 0.958 obtained as the ratio of experimental [89] and calculated frequency of the OH stretching mode in an isolated HOD molecule.

Figure A1 shows a $Q/Q_{0}$ ratio as a function of the OH mode frequency $\omega_{\mathrm{OH}}$, where *Q* is either $\mu^{\prime }$ or R and $Q_{0}$ denotes a value of *Q* for the OH mode of an isolated HOD molecule. One observes a weak dependence of R on $\omega_{\mathrm{OH}}$, in an agreement with results of refs. [79,85]. Consequently, the effect of environment on $\alpha^{\prime }$ was ignored and only the $\mu^{\prime }$ results were used to obtain the scale factor for $\chi^{\left(2\right)}$. A fit of the $\mu^{\prime }/\mu_{0}^{\prime }$ data with an ad hoc function

(A5) $$F\left(\omega \right)=a\ln(-b\left(\omega -\omega_{0}\right))$$

provides the sought frequency-dependent scale factor that is applied to the $\chi^{\left(2\right)}$ spectra reported in the paper. Values of the fit parameters with standard errors given in parentheses are a=6.4(0.9), $b=1.7\;\left(0.4\right)\cdot 10^{-3}$ cm, $\omega_{0}=4510\;\left(153\right)$ cm^−1^; the Pearson correlation coefficient is 0.92. An alternative scale factor based on results of Skinner and co-workers [79,80], was employed for correcting intensities of simulated VSFG spectra in refs. [42,90]. Figure S10 shows the $\mu^{\prime }/\mu_{0}^{\prime }$ ratio in the O–H stretching mode(s) for the H_2_O and HOD molecules. Except few points, the dependencies of the scaled dipole derivative on the OH mode frequency overlap with each other. Such a result indicates a weak influence of the intramolecular coupling on the quantity and thereby supports the validity of the local-mode approximation.
