# Supplementary material for: Structure and Nonlinear Spectra of the Basal Face of Hexagonal Ice: A Molecular Dynamics Study
Source: Molecules. 2025 Sep 4;30(17):3619. doi: 10.3390/molecules30173619 (PMC12430504; doi:10.3390/molecules30173619)
Supplement: Supplementary file 1 [file molecules-30-03619-s001.zip › molecules-3841136-supplementary.pdf]

Supplementary Materials: Structure and Nonlinear Spectra of the Basal Face of Hexagonal Ice: A Molecular Dynamics Study

Konstantin S. Smirnov [1*]

1. Structure of hexagonal ice Ih

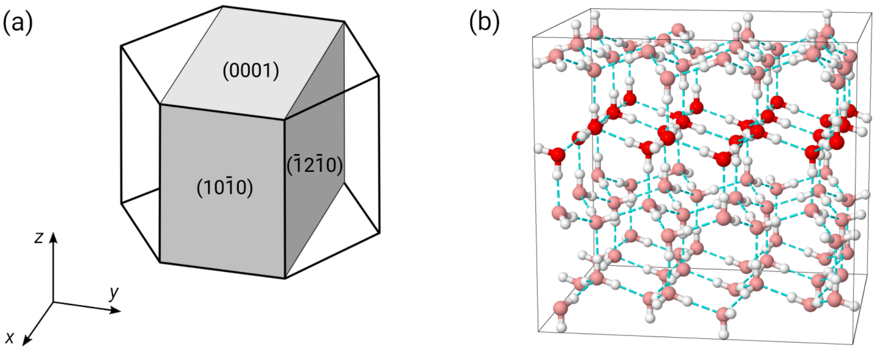

Figure S1. (a) Crystallographic cell of hexagonal ice Ih in the Cartesian frame of the simulation box shown as the shaded rectangular prism. The basal, 1st prismatic and 2nd prismatic faces are labeled with their Bravais-Miller indices, (0001), (10$\bar{1}$0) and ($\bar{1}$2$\bar{1}$0), respectively. (b) ice Ih structure. Atom colors: white–hydrogen, red–oxygen; oxygen atoms of one bilayer (BL) of the basal plane are highlighted with deep red color, the cyan dashed lines stand for H-bonds.

2. Structural characteristics of the ice Ih/air interface at $\Delta T = -35$ K

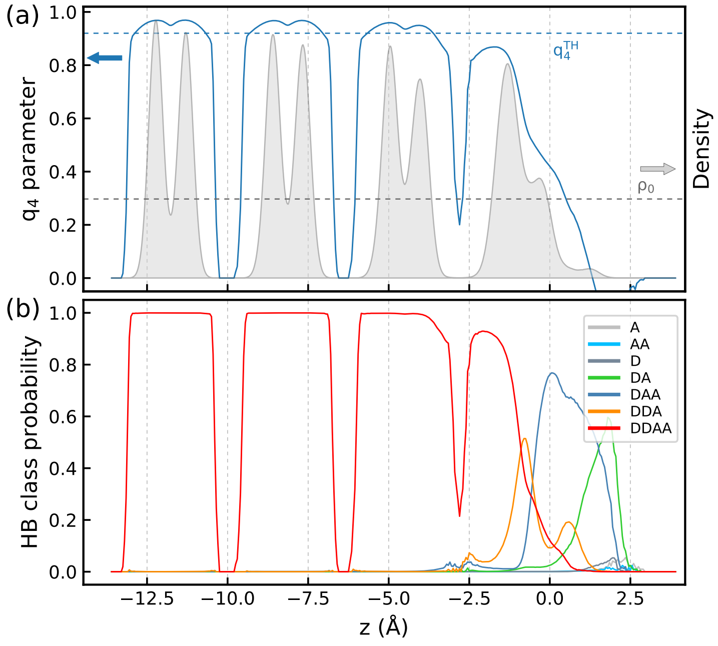

Figure S2. (a) z-profiles of water density ρ^* and of the q_4 order parameter, (b) z-profiles of H bonded species in the first four bilayers of the Ih/air interface at $\Delta T = -35$ K. The dashed horizontal lines in the panel (a) indicate the q_4^{TH} and $\rho^* = 1$ values.

3. Conditional probability maps of the orientation of water molecules in the bulk region and in the second bilayer

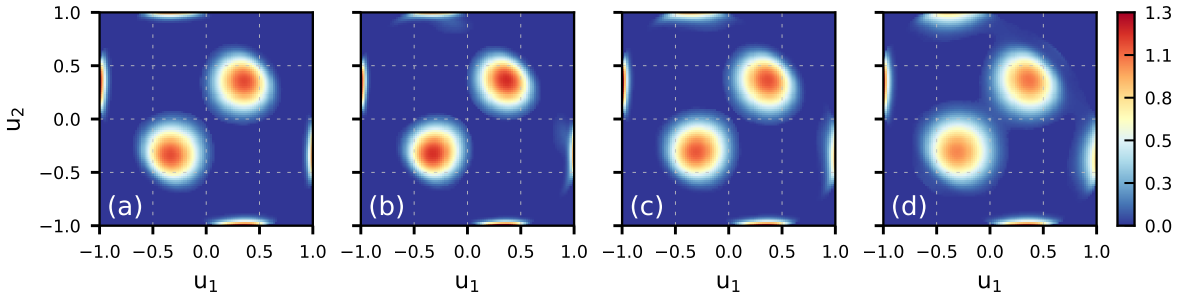

Figure S3. $D_{\mathcal{Z}}(u_1, u_2)$ maps for H_2O molecules. (a) bulk region of the ice Ih slab at $\Delta T = -5$ K, (b) the second bilayer (2BL) of the slab at $\Delta T = -65$ K, (c) 2BL at $\Delta T = -35$ K, (d) 2BL at $\Delta T = -5$ K.

4. Im[$\chi^{(2)}_{ssp}$] spectra as a function of probing depth

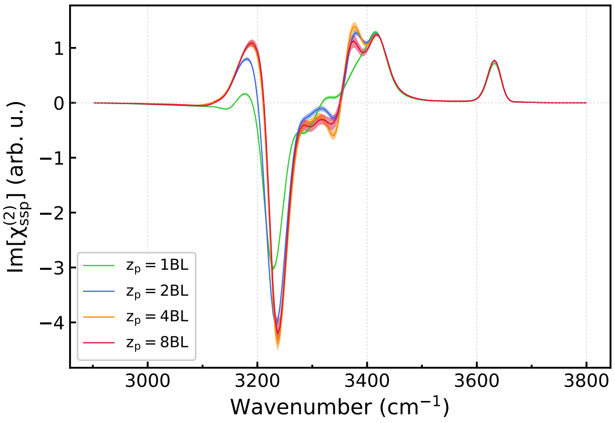

Figure S4. Im[$\chi^{(2)}_{ssp}$] spectra of the Ih/air interface at $\Delta T = -65$ K as a function of probing depth z_p. Shaded areas represent the statistical uncertainty.

5. Snapshots the two first bilayers of the ice/air interface

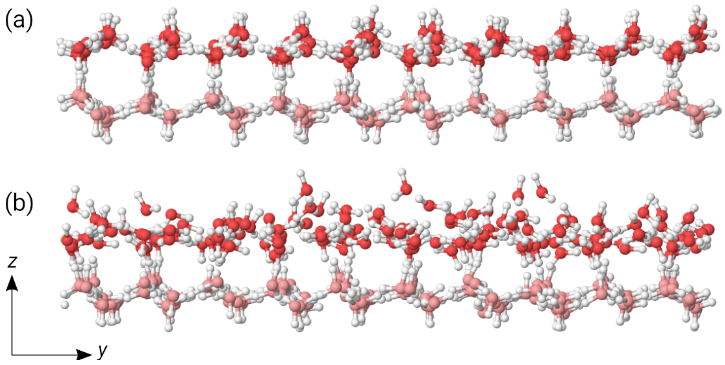

Figure S5. Snapshots of the two top bilayers of the Ih/air interface: (a) $\Delta T = -65$ K, (b) $\Delta T = -5$ K. The oxygen atoms of the first and second bilayer are shown with deep and pale red colors, respectively, and the hydrogen atoms are in white color.

6. Im[$\chi^{(2)}_{ssp}$] spectra of triply and doubly H-bonded surface water molecules

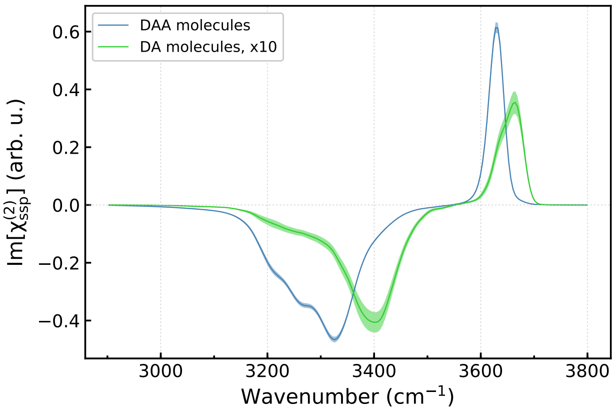

Figure S6. Im[$\chi^{(2)}_{ssp}$] spectra of DDA and DA molecules on the surface of the Ih/air interface at $\Delta T = -5$ K. Shaded areas represent the statistical uncertainty.

7. Oxygen–oxygen radial distrbution functions in the two first bilayers

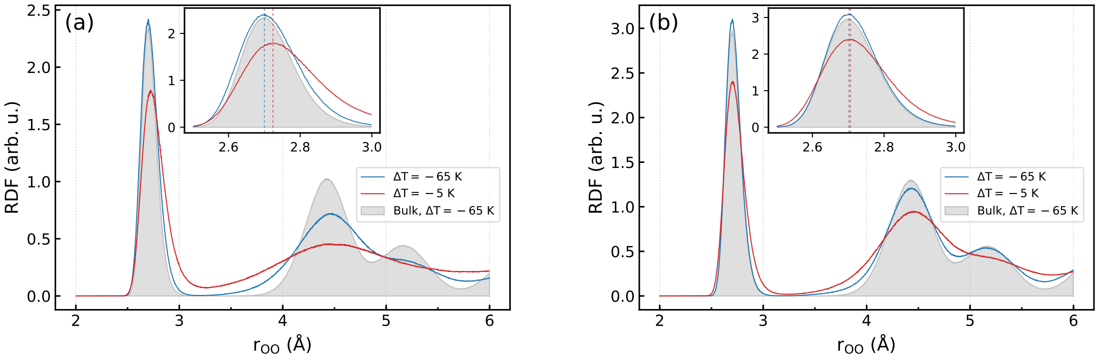

Figure S7. Radial distribution function (RDF) of the O–O atoms pair in the first (a) and second (b) bilayers of the Ih/air interface at $\Delta T = -65$ K and $\Delta T = -5$ K. The zoom shows the variation of the first peak position with temperature. The shaded contour in both panels shows RDF in bulk at $\Delta T = -65$ K.

8. Nonlinear spectra in the ppp polarization setting

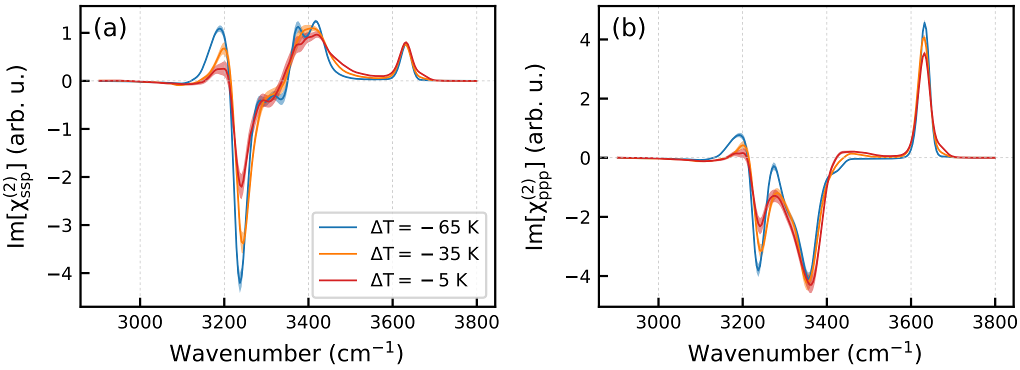

Figure S8. $\mathrm{Im}[\chi^{(2)}_{pqr}]$ spectra of the Ih/air interface at $\Delta T = -65$ K computed for different polarizations: (a) – ssp geometry, (b) – ppp geometry. Shaded areas represent the statistical uncertainty.

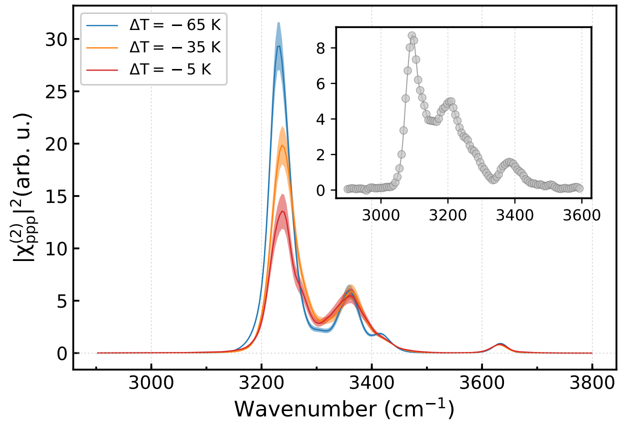

Figure S9. $|\chi^{(2)}_{ppp}|^2$ spectrum the Ih/air interface as a function of temperature. Shaded areas represent the statistical uncertainty. The inset shows the experimental *ppp* VSFG spectrum of the basal Ih surface at $\Delta T = -100$ K [81].

9. Dipole derivatives of H_2O and HOD molecules in the OH stretching mode

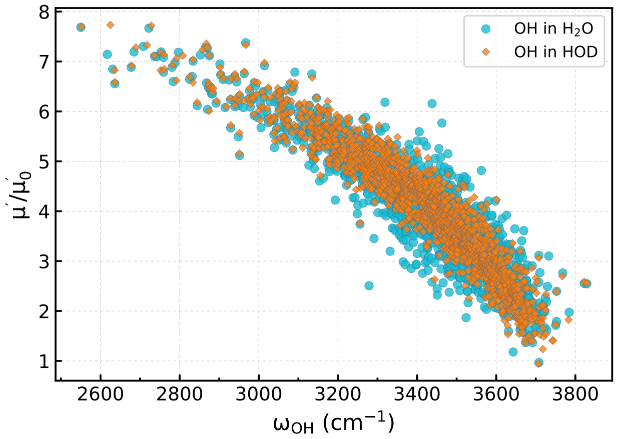

Figure S10. Scaled dipole derivative μ' / μ'_0 in the O–H stretching mode(s) of H_2O and HOD molecules, as a function of the mode frequency ω_{OH}.
